# Fabrication of High-Performance Asymmetric Supercapacitors Using Rice Husk-Activated Carbon and MnFe_2_O_4_ Nanostructures

**DOI:** 10.3390/nano13121870

**Published:** 2023-06-16

**Authors:** Faheem Ahmed, Shalendra Kumar, Nagih M. Shaalan, Nishat Arshi, Saurabh Dalela, Keun Hwa Chae

**Affiliations:** 1 Department of Physics, College of Science, King Faisal University, P.O. Box 400, Al-Ahsa 31982, Saudi Arabia; sjagdish@kfu.edu.sa (S.K.); nmohammed@kfu.edu.sa (N.M.S.); 2 Department of Physics, University of Petroleum & Energy Studies, Dehradun 248007, India; 3 Physics Department, Faculty of Science, Assiut University, Assiut 71516, Egypt; 4 Department of Basic Sciences, Preparatory Year Deanship, King Faisal University, P.O. Box 400, Al-Ahsa 31982, Saudi Arabia; nshastri@kfu.edu.sa; 5 Department of Pure & Applied Physics, University of Kota, Kota 324005, India; sdphysics@rediffmail.com; 6 Advanced Analysis & Data Center, Korea Institute of Science and Technology, Seoul 02792, Republic of Korea; khchae@kist.re.kr

**Keywords:** activated carbon, ferrites, high-performance electrodes, asymmetric supercapacitor, cyclic stability, 2D nanomaterials

## Abstract

To meet the growing demand for efficient and sustainable power sources, it is crucial to develop high-performance energy storage systems. Additionally, they should be cost-effective and able to operate without any detrimental environmental side effects. In this study, rice husk-activated carbon (RHAC), which is known for its abundance, low cost, and excellent electrochemical performance, was combined with MnFe_2_O_4_ nanostructures to improve the overall capacitance of asymmetric supercapacitors (ASCs) and their energy density. A series of activation and carbonization steps are involved in the fabrication process for RHAC from rice husk. Furthermore, the BET surface area for RHAC was determined to be 980 m^2^ g^−1^ and superior porosities (average pore diameter of 7.2 nm) provide abundant active sites for charge storage. Additionally, MnFe_2_O_4_ nanostructures were effective pseudocapacitive electrode materials due to their combined Faradic and non-Faradic capacitances. In order to assess the electrochemical performance of ASCs extensively, several characterization techniques were employed, including galvanostatic charge –discharge, cyclic voltammetry, and electrochemical impedance spectroscopy. Comparatively, the ASC demonstrated a maximum specific capacitance of ~420 F/g at a current density of 0.5 A/g. The as-fabricated ASC possesses remarkable electrochemical characteristics, including high specific capacitance, superior rate capability, and long-term cycle stability. The developed asymmetric configuration retained 98% of its capacitance even after 12,000 cycles performed at a current density of 6A/g, demonstrating its stability and reliability for supercapacitors. The present study demonstrates the potential of synergistic combinations of RHAC and MnFe_2_O_4_ nanostructures in improving supercapacitor performance, as well as providing a sustainable method of using agricultural waste for energy storage.

## 1. Introduction

Recent developments in renewable energy sources, portable electronics, and electric vehicles have increased the demand for efficient and sustainable energy storage systems. In order to meet this demand, companies are developing advanced energy storage technologies, such as supercapacitors, lithium-ion batteries, and hydrogen fuel cells. The energy density of these technologies is higher, the life cycle is longer, and they are more efficient than traditional storage methods. The ability of supercapacitors to store and release energy on demand makes them a reliable source of energy when required for long periods of time. This ensures a continuous energy supply and reduces fossil-fuel dependence [1]. In particular, supercapacitors have become an attractive candidate for high-power energy storage due to their fast charging and discharging rates, long cycle lives, and high power-densities [2]. Additionally, supercapacitors are safer than traditional batteries since they do not contain combustible materials and can be used in a wide range of applications. Electric vehicles benefit from their rapid acceleration and high power output, making them particularly suitable for use in electric vehicles [3]. However, developing supercapacitors with improved energy density remains a significant challenge. This challenge has prompted researchers to explore advanced electrode materials and innovative device configurations. Biomass-derived activated carbon (AC), with its cost-effectiveness, porous structure, and surface functionalities, is widely recognized as a suitable substrate for synthesizing different nanocomposites [4]. Activated carbon is a popular electrode material due to its high surface area, excellent electrical conductivity, and chemical stability. It is also non-toxic, making it an ideal material for use in renewable energy applications. This makes it an attractive choice for many applications such as supercapacitors, fuel cells, batteries, and solar cells [5]. Rice husk, for example, provide an environmentally friendly and cost-effective alternative to conventional carbon sources, contributing to waste valorization and resource utilization. Activated carbon is manufactured from rice husk for several reasons [6]. A large amount of rice husk is generated during rice processing, which is a common agricultural waste. The waste material is often burned or disposed of in landfills, causing pollution in the environment. Rice husk, however, can be converted into valuable activated carbon through a sustainable approach, reducing waste generation and minimizing the environmental impact. Rice husk-activated carbon has several desirable properties for use in supercapacitors [6]. The specific surface area of rice husk-activated carbon is typically hundreds to thousands of square meters per gram, providing abundant surface sites for storing charges [7]. Its large surface area facilitates efficient ion adsorption/desorption due to its high double-layer capacitance. Moreover, RHAC possesses a pore structure with micropores and mesopores. Its structure makes RHAC particularly useful for energy storage applications due to its ability to transport and diffuse ions. Furthermore, RHAC has excellent electrochemical stability and low internal resistance, making it suitable for high-power applications [7]. The presence of a porous network in the electrode allows ions to diffuse quickly and enhances charge-transport kinetics. The mesopores in particular improve access to the electrolyte, ensuring effective use of the active sites in the electrode. This results in high-power performance in supercapacitors by increasing charge storage abilities and reducing the internal resistance in the carbon-based electrodes [8].

Moreover, incorporating nanoscale materials into supercapacitor electrodes has shown great potential in enhancing their electrochemical performance. Spinel ferrites have been found to be a new class of low-cost materials applicable to a range of demanding applications, including photoelectrocatalytic materials and capacitive deionization. These materials have a variety of desirable properties, including high electrical and thermal conductivity, low cost, and high chemical stability. Furthermore, spinel ferrites have a wide range of potential applications in energy storage and conversion, water treatment, and other advanced technologies [9,10,11]. With their well-ordered Fe_2_O_4_ crystal lattice coupled with divalent metals such as Co, Mn, Zn, Ni, etc., the ferrites exhibit a multifarious chemical composition, synergistic physicochemical properties, and superior electrochemical properties. Consequently, they are ideal materials for synthesizing advanced nanomaterials. Energy storage, electrocatalysis, and sensing are among their potential applications [12,13]. In electrochemical applications, ferrites are potentially promising because of their high resistance to toxic chemicals, abundant viability, and high thermal stability. Transition metal oxides, such as MnFe_2_O_4_, have garnered significant interest due to their unique redox properties and high theoretical capacitance, high redox activity, abundant active sites, and good cycling stability. The pseudocapacitance of MnFe_2_O_4_ nanostructures provides higher energy storage capacity, enhanced rate capability, and enhanced cycling stability [12]. In addition, MnFe_2_O_4_ nanostructures have a large surface-to-volume ratio, which facilitates efficient charge transfer. As a result of the large surface area, redox reactions can occur at a greater rate, resulting in increased capacitance and energy storage. MnFe_2_O_4_ exhibits a unique crystal structure that enables facile ion diffusion and intercalation/deintercalation processes, facilitating pseudocapacitive charge storage mechanisms [14]. Due to these features, MnFe_2_O_4_ nanostructures are suitable as positive electrode materials for a variety of applications.

To achieve superior electrochemical performance, the supercapacitor must have an asymmetric configuration. The asymmetric design combines the advantages of positive and negative electrode materials by using different electrode materials. Combining the advantages of both electrodes, the asymmetric configuration enables a wider voltage window, a higher energy density, and a longer lifetime for the device [15]. With the successful integration of these materials, energy storage devices could become more efficient and sustainable. In addition to increasing the energy storage capacity and rate capability, the positive electrode provides pseudocapacitive behavior [16,17]. Meanwhile, the negative electrode simultaneously increases the double-layer capacitance, resulting in enhanced charge/discharge efficiency and higher specific capacitance. As a consequence of the asymmetric configuration, the supercapacitor has a wider operating voltage window. With different voltage ranges for the positive electrode and negative electrode, the device can maximize its overall energy density [18].

It was observed that by expanding the voltage window or lowering the scan rate, more energy can be stored in the cell and a wider range of applications can be accommodated [19]. However, a detailed study of ASCs composed of AC and ferrite-based electrodes has not been reported yet. In this study, rice husk-activated carbon and MnFe_2_O_4_ nanostructures are used as negative and positive electrode materials, respectively, to fabricate high-performance asymmetric supercapacitors. It is possible to increase the energy density, cycling stability, and overall electrochemical performance of supercapacitors by using these materials in combination. Rice husk-activated carbon and MnFe_2_O_4_ nanostructure electrodes are successfully integrated into asymmetric supercapacitors, which contribute not only to the advancement of energy storage systems, but also to sustainable energy management and waste management by efficiently utilizing a renewable energy source (rice husk). This research will open up new avenues for the development of efficient and sustainable energy storage devices for the integration of renewable energy, portable electronics, and electric vehicles.

## 2. Experimental Details

### 2.1. Materials

In this study, KCl, ethanol, acetone, ferric chloride hexahydrate (FeCl_3_·6H_2_O), MnCl_2_, sodium hydroxide (NaOH), methanol (CH_3_OH), 30% HCl, Nafion, and KOH were purchased from Sigma-Aldrich, St. Louis, MO, USA, while rice husk was purchased from the SDF chemicals, Gandhidham, Gujarat, India. Nickel foam, aluminum foil, and carbon paper were obtained from MTI, New York, NY, USA. All the materials were used as received without any further modification. The chemicals and reagents were analyzed for purity using analytical-grade instrumentation.

### 2.2. Preparation of Rice Husk-Activated Carbon (RHAC)

**Figure 1:**
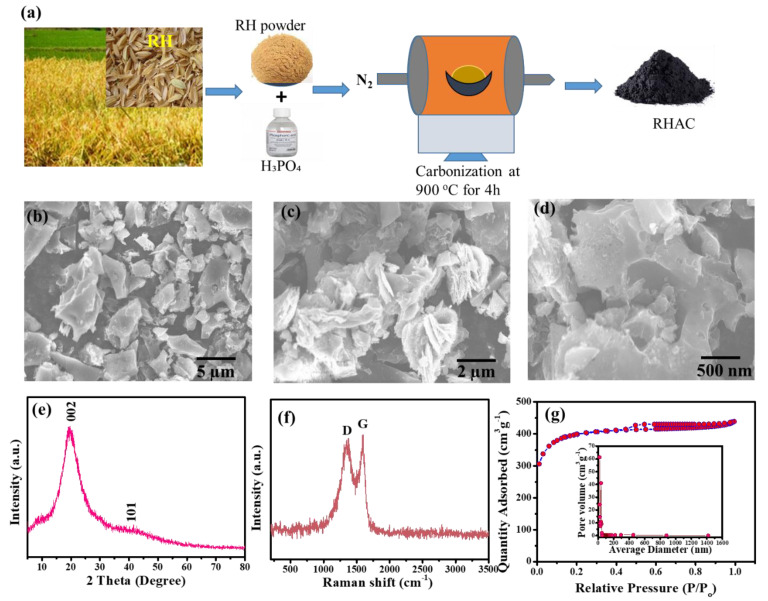
(**a**) Schematic diagram for the preparation of RHAC, (**b**–**d**) SEM images of RHAC, (**e**) XRD pattern of RHAC, (**f**) Raman spectrum of RHAC, and (**g**) N_2_ adsorption and desorption isotherm and pore size distribution of RHAC.

An electric grinder was used to grind the rice husk after it had been washed with DI water to remove dirt and other impurities. The ground material was then sieved using a sieve shaker to obtain the desired particle size of <200 microns. A single-step carbonization process was adopted to prepare the rice husk (RH)-derived activated carbon (RHAC) in a tubular furnace under an inert atmosphere. Briefly, 20 g of RH powder was mixed with an equimolar volume of H_3_PO_4_ and stirred for 2 h. After pretreatment, the biomass was dried, loaded into a furnace, and heated for 4 h at 900 °C at a rate of 5 °C/min in an N_2_ atmosphere. The carbonized samples were then washed with DI water to remove unreacted H_3_PO_4_ and/or adsorbed ions from the RHAC surfaces [20]. A final sample (RHAC) was dried in an oven at 110 °C overnight then stored in airtight sample bags for use in synthesizing electrodes. Figure 1a shows a detailed schematic for the preparation of RHAC.

### 2.3. Synthesis of 2D MnFe_2_O_4_

Microwave irradiation was used to synthesize 2D MnFe_2_O_4_ nanosheets. An equal volume of DI water and isopropyl alcohol solution (1:1) was mixed with 100 mL solution 0.2 M MnCl_2_.4H_2_O (obtained by dissolving 3.94 g manganese salt in 100 mL water) and 100 mL solution 0.8 M FeCl_3_.6H_2_O (obtained by dissolving 21.6 g iron salt in 100 mL water). MnFe_2_O_4_ gel was obtained after adding 100 mL solution 0.1 M NaOH to the Mn^2+^/Fe^3+^ solution dropwise and keeping it at 150 °C for 6 h. Ferrite-based intermediate metal hydroxides were formed by the ionic crosslinking of Mn^2+^/Fe^3+^ with OH^−^ ions present in the solution. The resultant gel was then calcined at 400 °C for 2 h followed by DI water washing and drying to obtain MnFe_2_O_4_ nanopowder [21]. A sealed container was used to store the final product, which was then used as an electrode material for the production of supercapacitors. The sealed container ensured that the product was safe from external contamination and that the composition of the material was kept consistent. The product was tested to verify its effectiveness as an electrode material. It was then used in the production of supercapacitors with excellent performance. 

### 2.4. Characterizations

The carbonized biomass (RHAC) and functional material (MnFe_2_O_4_ NSs) were thoroughly characterized by various analytical tools to examine the morphological features and chemical composition. The surface morphology and pore size were observed by scanning electron microscopy (SEM: JSM−7610F, JEOL, Tokyo, Japan) and a surface area analyzer (Model: 3Flex, Micromeritics, Norcross, GA, USA). X-ray diffraction (XRD) and Raman spectroscopy were also used to investigate the crystalline/amorphous nature of RHAC and its nanocomposite. XRD analysis was used to evaluate the crystalline structure of the developed materials at 40 kV and 40 mA at the 2θ ranges of 20–70°. X-ray photoelectron spectroscopy (ESCALab MKII, XPS) was used to determine the chemical composition and oxidation state of the MnFe_2_O_4_ NSs.

### 2.5. Electrochemical Analysis

Three electrodes were used to measure the electrochemical properties of MnFe_2_O_4_ NSs and RHAC. An Ag/AgCl reference electrode and a counter electrode were used in the cell. The working electrodes were developed by mixing 1 g of as-synthesized active material (whether RHAC or MnFe_2_O_4_) in 1 mL of Nafion solution, and then they were drop-casted on the commercially available Nickle foam. A vacuum oven was used to dry the electrodes overnight at 70 °C. The coated electrodes with the dimensions of 1 × 1 cm^2^ were then tested in an electrochemical cell to study the charge/discharge behavior, electrical resistance, and supercapacitor performance.

## 3. Results and Discussion

### 3.1. Morphological, Structural, and Compositional Analysis

FESEM was used to analyze the surface morphology of the as-synthesized RHAC and MnFe_2_O_4_ nanocomposites. The FESEM micrographs of RHAC at different magnifications are shown in Figure 1b–d. An FESEM image revealed a flaky structure with a porous and rough surface, while a higher magnification of 500 nm demonstrated an extended pore diameter, more visible voids, and a distorted porous surface, as shown in Figure 1c. This is due to the reason that the chemical (H_3_PO_4_) and thermal (900 °C) activation of the RH biomass resulted in the formation of mesoporous and microporous activated carbon, as also reported in the literature [22]. The surface characteristics, attachment of functional groups, and surface modification rely on various factors, namely, the activating agent’s properties, dosage, heating rate, and carbonization temperature [23]. These parameters play a crucial role in creating pores and facilitating the attachment of functional groups onto the surface of activated carbon (AC). As a result, the AC produced can be effectively utilized in diverse applications such as energy storage, catalysis, and environmental remediation. Furthermore, the physicochemical characteristics of the as-prepared RHAC were examined by using various analytical tools. Briefly, an X-ray diffraction (XRD) analysis for RHAC was performed to examine the crystalline or amorphous nature and instigate the phase purity of pristine RHAC (Figure 1e). A distinguished clear peak was observed at 2θ = 22.1°, ascribing to the (002) plane of graphitic carbon. Moreover, Raman spectroscopic analysis was adopted to examine the structural defects and the disorderliness in the crystal lattice of RHAC, as shown in Figure 1f. In brief, molecular vibrational studies provide insights into the defects and voids in the amorphous carbon structure. This information is reflected in the D band peak, which is used to determine the quality of the amorphous carbon. By using vibrational spectroscopy, molecules’ interactions and movements can be studied in more detail. This helps in understanding the amorphous carbon structure and its properties. Carbon-based nanostructured materials indicate the presence of well-organized graphitic layers as well as in-plane vibrations of sp^2^ aromatic carbon structures. Notably, the G band peak is an important indicator of the quality of the material and is used to determine its suitability for various applications [23]. A Raman spectrum of RHAC showed (see Figure 1f) characteristic D and G band peaks at 1425 cm^−1^ and 1682 cm^−1^. The relative intensity of the D and G bands is indicative of the degree of graphitization of the sample. The RHAC sample showed a ratio of 0.91, indicating a high degree of graphitization. The results were in accordance with the published literature on activated carbon derived from various biomass sources [6,24]. Additionally, the porosity of activated carbon derived from rice husk (RH) using H_3_PO_4_ activation was studied by N_2_ gas adsorption/desorption isotherms, as shown in Figure 1g. The results indicated that the activated carbon exhibited a type I desorption isotherm, suggesting it possessed a mesoporous structure. In the porous cavities, N_2_ gas uptake increased rapidly until the relative pressure reached 0.4, then it slowed down due to capillary condensation. The pore size distribution was found to be wide, with an average pore diameter of 7.2 nm. Furthermore, the BET surface area for RHAC was determined to be 980 m^2^ g^−1^. It also showed a considerable pore volume of 0.630 cm^3^ g^−1^, suggesting that it could be used as a supercapacitor electrode.

**Figure 2:**
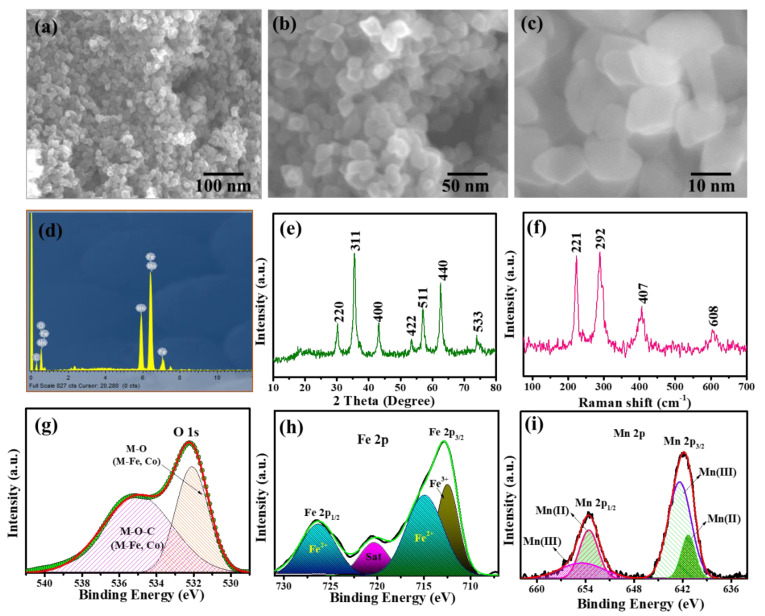
(**a**–**c**) FESEM images, (**d**) EDS spectra, (**e**) XRD pattern, (**f**) Raman spectra, (**g**) high-resolution O1s spectrum, (**h**) Fe 2p deconvoluted spectrum, and (**i**) high-resolution Mn 2p deconvoluted spectrum of MnFe_2_O_4_ nanoparticles.

Likewise, the morphological and structural features of spherically shaped 2D MnFe_2_O_4_ were also studied (Figure 2). The morphological features and the topographic aspects of MnFe_2_O_4_ were studied through FESEM. The micrograms presented the nanospheres as shown in Figure 2a–c, which are expected to be a promising electrode material for supercapacitor application due to the tuned morphology and enhanced charge storage capacity. The elemental composition was presented by energy dispersive spectroscopy (EDS) analysis. Figure 2d demonstrates the major elements detected in the EDS analysis of MnFe_2_O_4_. The results revealed that the MnFe_2_O_4_ comprises Mn, F, and O, while smaller peaks of C were also observed, which could be due to the carbon substrate used as a support for EDS analysis. In addition, XRD and Raman analysis was also conducted to assess the crystal lattice and structural disorderliness in the as-synthesized MnFe_2_O_4_ nanospheres. According to the diffractogram, the wider peaks range from 18° to 61.5°, corresponding to the (111), (220), (311), (400), (422), (511), and (440) crystal planes, respectively. MnFe_2_O_4_ exhibited a cubic spinel structure as described in JCPDS card no. 73–1964. In addition, Figure 2f shows the room temperature Raman spectrum of the MnFe_2_O_4_ nanoparticles. It is observed that MnFe_2_O_4_ exhibits four stretching vibrational modes in its spectrum [25]. The vibrational modes of the MnFe_2_O_4_ lattice show an intense band at 221 cm^−1^, while the O-H bond stretching vibrations appear at 292 and 608 cm^−1^. The Raman shift peaks at 407 and 608 cm^−1^ are caused by the E_g_ and T_2g_ vibrational modes, respectively [26].

To further investigate the chemical composition, oxidation states, and possible ionic valences present in the compounds, XPS analysis was conducted on MnFe_2_O_4_. The XPS spectrum of MnFe_2_O_4_ presented three main peaks that were attributed to O1s, Fe 2p, and Mn 2p as shown in Figure 2g–i, respectively. The deconvoluted spectrum of O1s shows four distinct peaks at 530.1, 531.2, 533.3, and 536.7 eV for MnFe_2_O_4_ (Figure 2g). A developed MnFe_2_O_4_ exhibits Fe-O, H-OH, Mn-O, and Fe-OH peaks, respectively. It was shown that oxygen vacancies significantly influence the magnetic interactions in MnFe_2_O_4_. Deconvoluted O1s displayed -O and -OH signals related to H_2_O bonded on MnFe_2_O_4_ ferrites [21,27]. There is evidence that water is adsorbed on the surface of the catalyst since the binding energy for the H_2_O peak (531.22 eV) is low. The presence of hydrogen-bonded water molecules is beneficial for the catalytic activity of MnFe_2_O_4_. The adsorbed water molecules help to reduce the activation energy of the reaction, increasing the rate of catalytic reactions. The XPS spectrum of Fe 2p_3/2_ (Figure 2h) revealed two distinct peaks associated with spin-orbital coupling, corresponding to Fe (II) and Fe (III) states. The Fe (II) peak was at a binding energy of 710.9 eV, while the Fe (III) peak was at a binding energy of 724.2 eV. This indicated that the sample contained both Fe^2+^ and Fe^3+^ ions [28]. This difference in binding energy was used to identify the two different states of iron in the sample. A low-intensity curve is observed in the Fe-deconvoluted spectrum. This low-intensity curve is due to the presence of Fe_2_O_3_ and FeO, which are commonly found as impurities in the nanocatalyst. A weaker intensity of the Fe-deconvoluted spectrum can be caused by these impurities. Additionally, the deconvolution of the Mn 2p core level spectra (Figure 2i) revealed four major peaks at 639.7, 641.1, 651.4, and 652.4 eV, confirming the Mn^2+^ state. The X-ray diffraction data also indicate that there were no other Mn valence states present and that Mn ions were in octahedral coordination with Mn^2+^. There are similar shoulder peaks and spin-orbital splitting energies observed of Mn 2p and MnO_2_. It appears that the Mn is oxidized with Mn^2+^ in octahedral coordination. A Mn ion is surrounded by six oxygen ions at equal distances. This is consistent with the observed spin-orbital splitting and binding energies of MnO_2_. The presence of Mn^2+^ and Fe^3+^ was further confirmed by XRD analysis [29]. As a result of the XPS analysis of the developed electrode material, it was confirmed that the MnFe_2_O_4_ contained a variety of compositions with mixed-valence heterostructures and hence could be a promising candidate for energy storage devices.

### 3.2. Electrochemical Characterization

**Figure 3:**
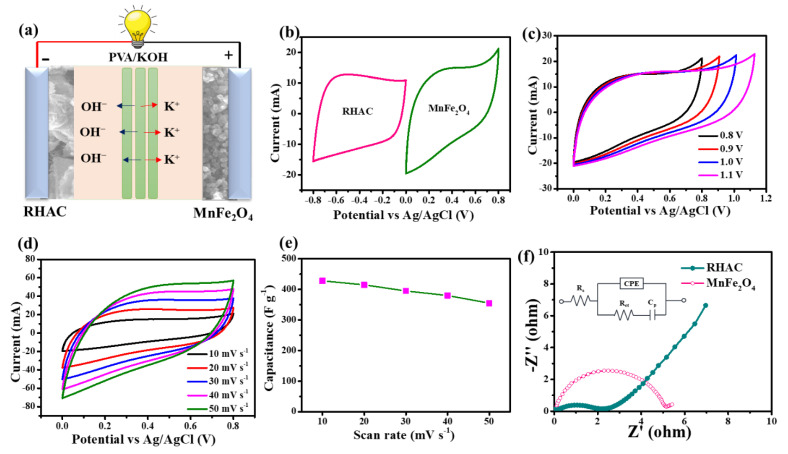
(**a**) ASC schematic diagram, (**b**) CV curves of MnFe_2_O_4_ electrodes and RHAC electrodes at a scan rate of 10 mV s^−1^, (**c**) CV curves of ASC devices in various potential windows at a constant scanning rate of 10 mV s^−1^ per second, (**d**) CV curves of the ASC device at various potentials (10–50) mV s^−1^, (**e**) specific capacitances of the ASC device at different potentials (10–50) mV s^−1^, and (**f**) EIS spectrums of the RHAC and MnFe_2_O_4_ electrodes.

A schematic of an asymmetric supercapacitor (ASC) constructed from RHAC and MnFe_2_O_4_ nanoparticles in a two-electrode system using PVA/KOH gel electrolytes is shown in Figure 3a. Furthermore, as-designed ASCs were electrochemically evaluated using platinum (Pt) and Ag/AgCl as counter and reference electrodes in 6 M KOH using a three-electrode system. In general, current–voltage relationships (CV) and electrochemical impedance spectroscopy (EIS) are used to determine the electrochemical properties of working electrodes. In CV measurements, the charge transfer rate is determined, whereas in EIS measurements, the charge transfer resistance is determined. These measurements are important for evaluating the electrochemical performance of the ASCs. The results can be used to optimize the design of the ASCs for improved performance. Both measurements are essential for understanding the electrochemical properties of working electrodes. Voltammograms of RHAC and MnFe_2_O_4_ nanoparticles are presented in Figure 3b,c at scan rates of 10 mV/s within potential ranges of −0.8 to 0 V and 0 to 0.8 V. The voltammograms show two distinct and clearly visible peaks for both RHAC and MnFe_2_O_4_ nanoparticles, indicating good electrochemical performance. The peaks also had moderate current densities indicating good conductivity. As indicated by their quasi-rectangular shapes, both materials showed electric double-layer capacitances (EDLCs). However, MnFe_2_O_4_ nanoparticles showed a higher specific capacitance owing to a combination of Faradic and EDLC (non-Faradic reactions). This was due to the greater surface area of the nanoparticles, which increased the number of active sites for Faradic reactions. The specific capacitance of the MnFe_2_O_4_ nanoparticles was two times higher than that of the graphene/MnFe_2_O_4_ nanocomposite. This suggests that MnFe_2_O_4_ nanoparticles are promising materials for high-performance supercapacitor applications. The applied voltage and scan rate have an impact on the specific capacitance of an electrochemical capacitor. The applied voltage and scan rate determine the rate at which ions move through the electrolyte and are adsorbed onto the electrodes. It was observed that by increasing the applied voltage from 0.8 V to 1.1 V, the specific capacitance increases (Figure 3c). This is because by increasing the voltage, pseudocapacitive materials may undergo faradaic reactions at the electrode–electrolyte interface and exhibit reversible redox reactions, allowing for additional charge storage beyond the electrostatic double-layer capacitance. Furthermore, the increased potential difference allows for a greater number of ions to participate in the faradaic reactions, leading to enhanced charge storage capacity. It is important to note that there are limits to the applied voltage that an electrochemical capacitor can withstand. Exceeding the maximum voltage limit can lead to electrolyte breakdown, electrode degradation, or even catastrophic failure. Therefore, the potential window for the designed supercapacitor must be optimized to enhance the electrochemical performance. In Figure 3d,e, scan rates are shown in relation to specific capacitance for the ASC system as designed. Electrochemical capacitors tend to have a lower specific capacitance at higher scan rates. Higher scan rates result in faster charging and discharging cycles during electrochemistry. During charging/discharging, ions diffuse between electrode materials and electrolytes. A higher scan rate reduces the effective capacitance because ions have less time to diffuse into and out of the electrode material. On the other hand, specific capacitance increases at lower scan rates. When scan rates are slower, ions have more time to diffuse into the electrode material, enhancing the charge storage capacity. Several factors can affect the relationship between scan rate and specific capacitance, including the electrode material, electrolyte composition, and capacitor design. Hence, the specific capacitance behavior concerning the scan rate may vary for different systems. Moreover, the electrical conductivity and resistivity of the developed materials also effect the ASC performance and can be reported in terms of electrical impedance spectroscopy (EIS). In Figure 3f, the Nyquist plot along with the electrical circuit for RHAC and MnFe_2_O_4_ nanoparticles is shown in the frequency range of 100 kHz to 0.01 Hz. The Nyquist plot of the electrodes at different frequencies shows semi-circle profiles and straight inclined lines. A rapid diffusion of ions is observed in these profiles, as well as a double-layer capacitance with a charge resistance (Rct). The capacitance obtained was a few microfarads, indicating that the RHAC and MnFe_2_O_4_ nanoparticles are effective electrolyte materials. The charge-transfer resistance (Rct) of the electrodes was observed to decrease with increasing frequency. This indicates good electrochemical kinetics of the electrodes [30]. The R_ct_ values estimated through electrochemical impedance spectroscopy (EIS) for the RHAC and MnFe_2_O_4_ were 2.1 and 4.2 Ω, respectively. These values are lower compared to previously reported carbon-based electrodes [30,31,32]. The smaller R_ct_ values indicate improved charge storage properties and lower internal resistance in the electrodes. Hence, the as-synthesized electrodes could be promising candidates for asymmetric supercapacitors.

### 3.3. Asymmetric Supercapacitor Performance

**Figure 4:**
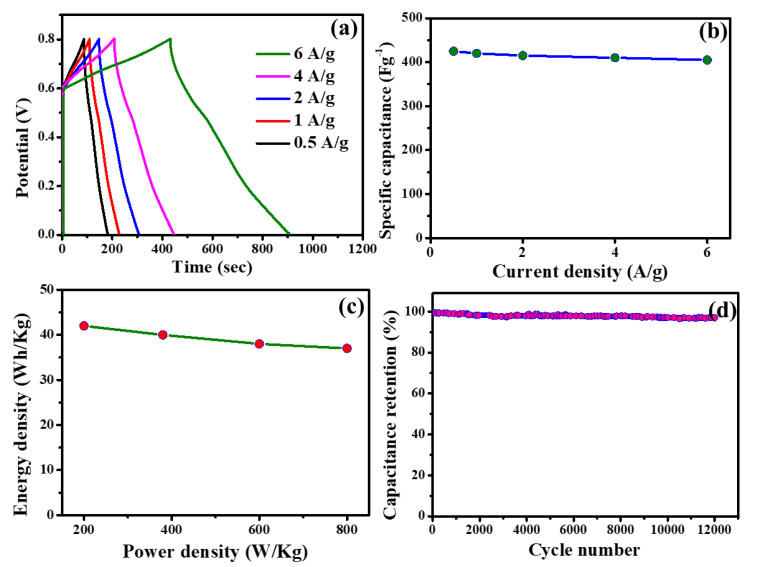
(**a**) GCD curves of the ASC device at different current densities (0.5 to 6 A/g), (**b**) specific capacitance of the ASC device at different current densities (0.5 to 6 A/g), (**c**) Ragone plots (energy density versus power density) of the ASC device, and (**d**) cycling stabilities of the ASC device during 12,000 GCD cycles at the current density of 6 A/g.

Figure 4a shows the galvanostatic charge–discharge curves for the electrodes in the asymmetric supercapacitor device at different current densities of 0.5, 1, 2, 4, and 6 A/g. The GCD (galvanostatic charge–discharge) curve typically starts from a non-zero voltage, such as 0.6 V, in order to avoid any initial transient effects or artifacts that may occur at very low voltages. This initial voltage value allows the system to reach a steady state before the actual measurement of capacitance begins. The capacitance calculation based on the GCD curve is typically performed by analyzing the voltage–time data obtained during the discharge portion of the curve. The capacitances of the devices increased with increasing current density, according to the results. It was found that at 0.5 A/g, the highest specific capacitance of 420 F/g was obtained, which was five times higher than at 6 A/g. These results demonstrate the device’s potential for high-power applications. Based on the GCD data, specific capacitances of ASC devices were calculated at various current densities. Ideally, the device should be tested to determine whether it can store and deliver large amounts of energy at high power-density and is therefore suitable for a wide range of energy storage applications. Using current densities of 0.5, 1, 2, 4, and 6 A/g, ASC displayed specific capacitances of 420, 418, 416, 408, and 400 F/g, respectively (Figure 4b). It is important to note that the capacitance calculated from the GCD curve represents the overall capacitance of the system under the specific experimental conditions. Different charging and discharging curves may arise due to various factors such as the electrode properties, electrolyte composition, and measurement setup. However, the capacitance calculated from the GCD curve provides a useful measure of the overall performance of the system in terms of its ability to store and release charge. As-designed ASC devices showed higher specific capacitances than carbon-based symmetrical ASC devices. In addition, the higher current densities did not result in a noticeable difference in capacitance values. Therefore, the designed ASC can serve as an energy storage device. According to Figure 4c, ASC performance was also evaluated based on the relationship between energy and power densities. An energy density of 40 Wh/kg was achieved at a power density of 400 W/kg, which is twice that reported for carbon-based symmetric SCs [6]. These results demonstrate that ASCs are promising for high power- and energy-density applications. Furthermore, the ASCs exhibited excellent cycling stability and can be easily scaled up to meet the requirements for energy storage applications. The energy density was maintained by increasing the power density to 800 W/kg. Furthermore, a cycle test at a current density of 6 A/g was conducted to evaluate the long-term performance of the ASC. In Figure 4d, the retention of capacitance (%) is shown as a function of the number of cycles. Although the ASC went through 12,000 GCD cycles, it retained its capacitance at ~98%. This indicates that the ASC has excellent cycling stability and can be used in various applications. Moreover, the ASC showed a high power-density and excellent cycling stability, which demonstrates its potential as an energy storage device. The results of this study suggest that the ASC can be a promising alternative to existing energy storage devices due to its high power-density and excellent cycling stability. Further research on the ASC is needed to explore its full potential. Its high specific capacitance, admirable rate performance, and virtuous cyclic stability indicate that the as-designed ASC exhibited superior electrochemical performances, attributing higher power-densities with minimal energy losses, and hence could be effectively employed for energy storage applications. 

## 4. Conclusions

In summary, asymmetric supercapacitors fabricated from rice husk-activated carbon and MnFe_2_O_4_ nanostructures hold tremendous promise for the development of advanced energy storage systems. This approach offers several advantages, such as low cost, abundance, and exceptional electrochemical properties, including the integration of rice husk-activated carbon as the negative electrode material. Structural studies confirmed the formation of activated carbon by the presence of carbon peaks in the XRD and Raman studies; however, MnFe_2_O_4_ exhibited a cubic spinel structure. Morphological features using SEM and TEM analyses show that activated carbon has a sheet-like structure, while spherically shaped morphology was observed for observed for MnFe_2_O_4._ Electrochemical studies show that charge/discharge efficiency and capacitance are both increased by the activated carbon’s high specific surface area, porosity, and efficient ion diffusion. In addition, MnFe_2_O_4_ nanostructures showed pseudocapacitance behavior and may be an effective anode material for asymmetric supercapacitors. Consequently, the developed electrodes have higher specific capacitances of 420 F/g, higher current densities, lower internal resistance, and minimal energy losses even after 12,000 cycles (at a current density of 6 A/g), which significantly improves the overall performance of the asymmetric supercapacitor, contributing to a higher energy storage capacity and cycling stability. These results demonstrate that MnFe_2_O_4_ nanostructures in combination with activated carbon are promising materials for supercapacitor applications, as they can store large amounts of energy (energy density of 40 Wh/kg) and have long-term cycling stability. This makes them ideal candidates for energy storage and conversion applications, such as in electric vehicles and renewable energy systems.

## Data Availability

Available on request.
